# Impact of COVID-19 on the 937 Telephone Medical Consultation Service in Saudi Arabia

**DOI:** 10.1155/2022/4181322

**Published:** 2022-01-18

**Authors:** Najla J. Alhraiwil, Sinaa Al-Aqeel, Amjad F. AlFaleh, Alaa A. AlAgeel, Mostafa A. AlAbed, Walid A. Al-Shroby

**Affiliations:** ^1^Evaluation & Impact Measurement Unit, Deputyship of Public Health, Ministry of Health, Saudi Arabia; ^2^Clinical Pharmacy Department, College of Pharmacy, King Saud University, Saudi Arabia; ^3^General Directorate of Medical Consultations, Deputyship of Public Health, Ministry of Health, Saudi Arabia; ^4^Public Health & Community Medicine Department, Faculty of Medicine, Beni-Suef University, Egypt

## Abstract

The impact of COVID-19 on healthcare services has been profound. One major impact has been underutilization of traditional healthcare services by patients. In 2020, the Saudi Ministry of Health (MoH) started offering general COVID-19 enquiries, education, and medical and psychological consultations around the clock via their 937-Call Center. Given this major change, there was a need to understand the impact of the COVID-19 pandemic on Call Center services, specifically medical consultations, to suggest future recommendations for patient care optimization. This descriptive study analyzed routinely collected data on the 937-Call Center service between March 2020 and September 2020. Data were reviewed, coded, verified, and analyzed using SPSS v22. There was a 296% increase in the number of calls received by the 937-Call Center in 2020 compared to the same period in 2019. The majority of calls received in 2020 were general medical enquiries (98.41%), but about three million COVID-19-specific enquiries were also received in 2020. The increased number of calls was managed by accepting volunteers to handle calls: an average of 236 volunteers per month, handling about 20% of the total call volume. The majority of volunteers were physicians but with the presence of pharmacists, psychologists, and specialized healthcare workers such as nutritionists. Utilization of the 937-Call Center increased during the COVID-19 pandemic, suggesting that it has been an effective strategy for combatting the COVID-19 pandemic in Saudi Arabia. Further research is recommended to investigate the impact of COVID-19 on public awareness of the 937-Call Center and other health-related mobile apps.

## 1. Introduction

The impact of COVID-19 on healthcare services has been profound. One major impact has been the underutilization of healthcare services by patients. For example, in the US, a decline in the use of outpatient care peaked in April 2020 [1], and similarly, there was a steep decline in consultation rates and significant reductions in referrals, tests, new prescriptions, and immunizations in England [2]. A UK-based study reported reduced secondary mental health service utilization both before and during lockdown during the COVID-19 pandemic [3]. A systematic review of 81 studies from 20 countries reported a median 37% reduction in services overall (interquartile range -51% to -20%), comprising median reductions of 42% (-53% to -32%) for visits, 28% (-40% to -17%) for admissions, 31% (-53% to -24%) for diagnostics, and 30% (-57% to -19%) for therapeutics [4].

In response, there were widespread switches to predominantly remote counseling. A US report estimated a 154% increase in telehealth visits during the last week of March 2020 compared to the same period in 2019, most likely due to pandemic-related telehealth policy changes and public health guidance [5]. As a result, evidence started to accumulate on the role of telehealth services for preventing, diagnosing, treating, and controlling diseases during the pandemic in different parts of the world [6].

The World Health Organization (WHO) defines telehealth, including telemedicine, as “the delivery of healthcare services, where patients and providers are separated by distance. Telehealth uses information communication technologies for the exchange of information for the diagnosis and treatment of diseases and injuries, research and evaluation, and for the continuing education of health professionals” [7].

Telehealth is one component of e-health, defined by the WHO as “the cost-effective and secure use of information communication technologies (ICT) in support of health and health-related fields including healthcare services, health surveillance, health literature, and health education, knowledge, and research.” Another definition of e-health is “an emerging field in the intersection of medical informatics, public health, and business referring to health services and information delivered or enhanced through the Internet and related technologies” [8].

The Saudi Ministry of Health (MoH) has long embraced telehealth, and first established communication and public telephone counseling in April 2004. A formal “e-health” strategy was then implemented in 2008 after the provision of financial support. This facilitated the launch of the 937-Call Center service in May 2013 for urgent reports and inquiries, especially for the MERS virus. The service was later supported by the launch of several mobile apps [9]. For instance, the “*Sehha App”* delivers teleconsultations through live video chat and text or voice massaging to provide health assessment and guidance on maintaining good health [10]. According to 2020 Saudi MoH data, the *“Sehha App”* has been downloaded to smart phones 1,551,247 times and has provided 1,284,824 medical consultations [11].

Another example is the “*Sehhaty App*,” which was launched to provide access to health information such as vital sign updates, tracking prescribed medicine, retrieving and sharing sick leave, and promoting a healthy lifestyle. Moreover, a mobile app- and web-based application called “*Mawid*” was launched in 2018 to provide access to the national central healthcare appointment gateway to enable patients to book their appointments at Saudi MoH Services.

These apps were updated in response to the COVID-19 pandemic by introducing a symptom checker to enable people with suspected COVID-19 to directly book testing appointments at dedicated COVID-19 clinics. Another app launched during the pandemic was “*Tawakkalna*,” which facilitates the electronic issue of movement permits during curfews and shows whether a person has COVID-19 and has been in contact with an infected individual and to confirm if the user has taken the COVID-19 vaccine. The “*Tabaud App*” enables users to receive direct and proactive notifications in case of contact with any registered and confirmed cases of COVID-19. Finally, the “*Tetamman App*” provides guidance for individuals who are in home isolation or quarantine. For full details of the telehealth applications available in Saudi Arabia during the COVID-19 pandemic, we refer readers to Hassounah et al. [12].

This paper focuses on 937-Call Center service. It is an existing telecommunication system service with a simple engineering architecture. The service use phone calls and specific software to record information gathered from the patients and outcomes of the consultation. The 937-Call Center provides medical consultations with qualified doctors, appointment reservations at MoH primary health centers, smoking cessation clinic appointments, and technical support for MoH e-applications. It also provides other services including medical referrals and treatment requests, complaints and reporting, receiving suggestions and creative ideas, general inquiries, and transaction follow-ups [13].

In 2020, the Call Center started offering services for general COVID-19 enquiries and providing instructions and medical and psychological consultations on all aspects of COVID-19. Through its various channels such as telephone, social media, email, electronic applications, and instant chat on the ministry's website, the 937-Call Center operates 24 hours a day, seven days a week. The center employs nearly 2,000 staff (physicians and customer service specialists) to provide the services. During the pandemic, physicians and psychologists volunteered at the service. A WhatsApp service for the 937-Call Center was launched in May 2020 to provide advice and education on COVID-19 as well as the Sign Language Application “*Eshara*” to provide the hard of hearing with services and information. All 937-Call Center services are provided free of charge to citizens, residents, and visitors [14].

There is a need to understand the impact of the COVID-19 pandemic on 937-Call Center services, specifically medical consultations, to suggest future recommendations for patient care optimization in any future waves of COVID-19 or infectious diseases. We hypothesized that COVID-19 increased the number of calls received by the 937-Call Center for medical consultations for two reasons: first, lockdown restrictions meant that patients used remote medical consultations more than before the pandemic; and second, the psychological and physical impact of COVID-19 might have increased the number of individuals requiring medical consultations, recognizing that there were many campaigns during the pandemic to promote the services.

Therefore, the study objectives were to (i) identify the total number of medical consultation calls to the 937-Call Center in 2019 and 2020, (ii) differentiate the types of enquiries for medical consultations received in 2020 (general medical consultations, pharmaceutical consultations, psychological consultations, poison consultations, dental consultations, nutrition consultations, type 1 diabetes consultations, and smoking cessation consultations), and (iii) identify the total number of calls received in 2020 in relation to COVID-19: COVID-19 general enquiries, COVID-19 symptoms or consultation, or infection with COVID-19.

## 2. Material and Methods

This descriptive study analyzed data routinely collected on 937-Call Center services from March to September 2020 and compared it to the same period in 2019. The only data considered were medical consultation calls, which did not include other services such as appointment booking or complaints. The data were retrieved from the General Directorate of Medical Consultations.

### 2.1. Statistical Analysis

Data were reviewed, coded, verified, and analyzed using SPSS v22 (IBM Statistics, Armonk, NY). Means and standard deviations (SD) were used to describe continuous data. Number (*N*) and percentages were used to describe categorical data. No comparative analysis was performed due to the descriptive nature of the study.

### 2.2. Ethical Considerations

This study was conducted in accordance with the Declaration of Helsinki and all applicable local regulations. The Central IRB of the Ministry of Health, Riyadh, Saudi Arabia (National Registration Number: H-01-R-009) approved and judged the study protocol as exempt from full review because it involved collection and analysis of existing data recorded by the investigators in such a manner that subjects could not be identified either directly or through identifiers linked to the subjects. Study data were stored securely and were only accessible by the research team.

## 3. Results

**Figure 1:**
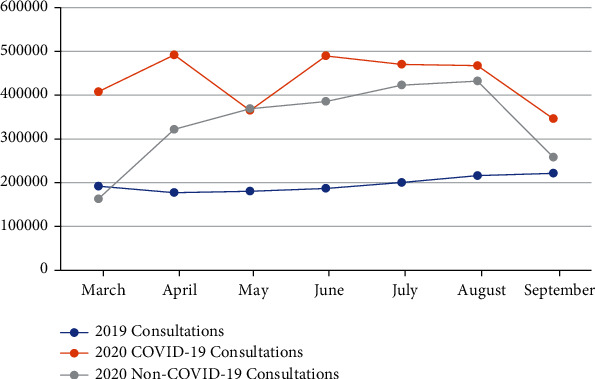
Number of COVID-19 consultations received by the 937-Call Center during 2020 compared to total calls received during 2019 (March-September).

1,375,071 and 5,446,275 calls were received between March and September in 2019 and 2020, respectively, representing a >296% increase. The majority of calls received during 2020 were related to general medical enquiries (98.41%), with pharmaceutical consultations and psychological consultations accounting for 45,295 and 35,119 calls, respectively (Table 1). It is also worth mentioning that call categorization started in 2020; therefore, it would be hard to compare the numbers with any 2019 data. On the other hand, there were approximately three million calls related to COVID-19 during 2020 with an average of 434,155 consolations per month (Figure 1).

**Figure 2:**
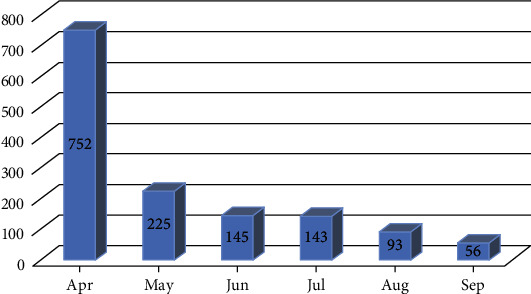
Number of volunteers joining the 937-Call Center between April^∗^ and September 2020. ^∗^Volunteers joined in March, but the official count of volunteers started on April, which includes both months.

The increased number of calls received by the 937-Call Center resulted in an increase in staffing, including by volunteers (Table 2). Volunteers did not answer calls in 2019, so represented a new division of the service added during the pandemic. Volunteers joined the service in March 2020 and began giving consultations alongside staff members, but they were officially registered in the MoH volunteering platform in April. The number of volunteers who started during the first month peaked at over 500 and then dropped to less than 100 in later months, resulting in an average of 236 volunteers per month depending on the call volume during the period (Figure 2).

## 4. Discussion

The results of this study confirm our hypothesis that the utilization of the 937-Call Center services increased during the COVID-19 pandemic, consistent with other international research reporting an increase in alternative healthcare delivery methods.

Alkhashan et al. [15] conducted an online questionnaire of 937-Call Center users (*n* = 5,005) and healthcare providers working at the 937-Call Center and the *Seha App* (*n* = 100) in 2018. The 937 Medical Consultation Call Center between January and June 2018 received over half a million calls (525,379), and 94,147 people around Saudi Arabia sought medical advice through the *Seha App* during the same period.

Al-Rays et al. [16] used an online questionnaire to measure the level of awareness and utilization of the 937-Call Center services. A total of 1,303 questionnaires were completed, 601 (46.1%) respondents reported that they were aware of the service, but only 258 (19.8%) respondents reported that they used the service. It is uncertain when this study was conducted, so it is not generalizable to the COVID-19 pandemic. In addition, an online survey of 781 participants in 2018 showed that half of respondents had never used the *Seha App* nor used a phone to seek medical advice [17]. But both survey samples were predominately females raising a question about the generalizability of their conclusions.

An increase in call volumes is to be expected during times when access to medical services is restricted or new health policies are implemented. The increased number of calls received by the 937-Call Center during 2020 more than doubled (142%) the number of employees dealing with public medical inquiries compared to 2019. This was not only due to the restrictions imposed by the government during the pandemic but also due to MoH campaigns prompting the use of e-health through the multiple channels providing services, i.e., strong advocacy to support e-health in an era of advanced technologies.

It is worth noting that although specialists and residents handled almost the same number of calls (Table 3), residents represented 30% of the total staff while specialists represented only about 13% of the total. There is no clear explanation for this difference. On the other hand, over 50% the total number of staff between March and September 2020 were volunteers; however, they only handled about 20% of calls, perhaps because most volunteers only volunteered for short periods of time. Nevertheless, volunteering made an important contribution to a very resource-stretched service, since the 20% of medical consultations handled by volunteers during the peak of the pandemic would have saved the Saudi MoH about 18% of the service cost had the volunteers been employed as full-time general physicians.

Future research should examine the perceived usefulness of the 937-Call Center and other health-related mobile apps and the degree of users' trust in these services after the COVID-19 pandemic. Previous local research has shown that reasons for reluctance to use telemedicine included being unable to know the doctor at a personal level (30%), a lack of trust in the technique (29%), and a lack of awareness about any benefits of telemedicine (28%) [15]. Other research has also shown an impact of perceived usefulness and perceived ease of use on attitudes of Saudi patients towards e-health services [18]. Alaboudi et al. [19] reported that the top barriers to adopting and implementing telemedicine by decision-makers in Saudi Arabia were (i) the availability of adequate sustainable financial support to implement, operate, and maintain the telemedicine system; (ii) ensuring conformity of telemedicine services with core mission, vision, needs, and constraints of the healthcare facilities; and (iii) reimbursement for telemedicine services.

## 5. Conclusion

The results of this study confirm that the utilization of the 937-Call Center increased during the COVID-19 pandemic, making the service one of the best strategies to combat the COVID-19 pandemic in Saudi Arabia. Volunteering should receive further investment through training and empowerment to increase efficiency and to save resources. Further research is required to investigate the impact of COVID-19 on awareness about the 937-Call Center and other health-related mobile apps.

## Figures and Tables

**Table 1:** The number and types of consultations received by 937-Call Center service in 2020.

Type of medical consultation inquiry	Start date^∗^	End date^∗∗^	Number of consultations (%)
General medical consultations	01/01/2020	21/12/2020	7,093,986 (98.41)
Pharmaceutical consultations	13/06/2020	21/12/2020	45,295 (0.63)
Psychological consultations	12/04/2020	21/12/2020	35,119 (0.49)
Poison consultations	01/01/2020	21/12/2020	18,428 (0.26)
Dental consultations	13/06/2020	21/12/2020	20,834 (0.29)
Nutrition consultations	13/06/2020	21/12/2020	12,196 (0.17)
Type 1 diabetes consultations	05/04/2020	21/12/2020	3,549 (0.05)
Smoking cessation consultations	16/08/2020	21/12/2020	2,673 (0.04)
Business support consultations^∗∗∗^	16/08/2020	21/12/2020	443 (0.01)
Total			7,232,523 (100)

^∗^When data first collected; ^∗∗^data collection end date; ^∗∗∗^a service started during the pandemic to target the business sector to provide COVID-19-related consultations.

**Table 2:** Change in the total number of staff and volunteers working in the 937-Call Center in 2019 and 2020 between March and September.

Staff category	2019 *n* (%)	2020 *n* (%)
Consultants	82 (15.76)	41 (1.53)
Specialists	237 (45.57)	352 (13.15)
Residents	154 (29.61)	828 (30.95)
Pharmacists	47 (9.03)	40 (1.49)
Volunteers	0 (0.00)	1414 (52.85)
Total	520 (100)	2675 (100)

**Table 3:** Change in percentage of calls received by each category of staff working in the 937-Call Center between 2019 and 2020 (March to September).

Staff category	Percentage of calls 2019	Percentage of calls 2020
Consultants	7.57%	2.36%
Specialists	53.53%	37.46%
Residents	27.25%	38.28%
Pharmacists	11.54%	1.62%
Volunteers	0.11%	20.28%
Total	100%	100%

## Data Availability

The data that support the findings of this study are available from the General Directorate for Medical Consultations, Deputyship of Public Health, Saudi Ministry of Health. Restrictions apply to the availability of the raw data, which were used under license for this study.
